# IgE reactivity to fish allergens from Pacific cod (*Gadus macrocephalus*) in atopic dogs

**DOI:** 10.1186/s12917-020-02559-1

**Published:** 2020-09-16

**Authors:** Ichiro Imanishi, Jumpei Uchiyama, Keijiro Mizukami, Junichi Kamiie, Keigo Kurata, Keita Iyori, Masato Fujimura, Kuniyoshi Shimakura, Koji Nishifuji, Masahiro Sakaguchi

**Affiliations:** 1 Laboratory of Veterinary Microbiology I, School of Veterinary Medicine, Azabu University, 1-17-71 Fuchinobe, Chuo-ku, Sagamihara, Kanagawa 252-5201 Japan; 2 Department of Microbiology, Kitasato University School of Medicine, Sagamihara, Kanagawa Japan; 3 Laboratory of Veterinary Pathology, School of Veterinary Medicine, Azabu University, Sagamihara, Kanagawa Japan; 4 Institute of Tokyo Environmental Allergy, ITEA Inc, Bunkyo-ku, Tokyo Japan; 5 Vet Derm Tokyo, Laboratory and Clinical Dermatology Services, Kouto-ku, Tokyo Japan; 6 Fujimura Animal Hospital, Minoo, Osaka Japan; 7 Department of Food Science and Technology, Tokyo University of Marine Science and Technology, Minato-ku, Tokyo Japan; 8 Laboratory of Veterinary Internal Medicine, Faculty of Agriculture, Tokyo University of Agriculture and Technology, Fuchu, Tokyo Japan

**Keywords:** Fish allergy, Atopic dermatitis, Animal model, Immunoglobulin E, Allergen component

## Abstract

**Background:**

IgE reactivity to fish allergens in atopic dogs, which are used as models for food allergy, has not been elucidated to date. We investigated IgE reactivity to crude extracts and purified allergens derived from the Pacific cod (*Gadus macrocephalus*) in atopic dogs to identify the allergenic proteins of cod.

**Results:**

The levels of specific IgE to crude cod extracts were measured in the sera of 179 atopic dogs, including 27 dogs with cod allergy, using enzyme-linked immunosorbent assay (ELISA). Specific IgE to crude cod extracts were present in 36 (20%) of the 179 atopic dogs and in 12 (44%) of the 27 dogs with cod allergy. The allergens in crude cod extracts were analyzed by ELISA, immunoblotting, and liquid chromatography-tandem mass spectrometry. In allergen component analysis, IgE reactivity to tropomyosin and enolase was observed in the sera of dogs with cod allergy. IgE reactivity to parvalbumin, collagen, and tropomyosin was evaluated using the sera of atopic dogs that tested positive for specific IgE to crude cod extracts. Among the 36 dogs with IgE reactivity to crude cod extracts, 9 (25%), 14 (39%), and 18 (50%) dogs tested positive for specific IgE to parvalbumin, collagen, and tropomyosin, respectively.

**Conclusions:**

The IgE reactivity to cod allergens observed in dogs was similar to that in humans, and this finding further supports the use of atopic dogs with fish allergy as a model for fish allergy in humans.

## Background

The prevalence of fish allergy, which affects approximately 0.2% of the 7.8 billion global population [1], is over ten times higher in geographic regions where fish is an essential dietary component, such as Japan [2, 3]. Fish allergy is typically known to persist as a life-long condition in contrast to other food allergies [3]. Since clinical cross-reactivity to different fish species is a widely observed characteristic in fish allergy, affected individuals have to compulsorily avoid the consumption of any fish species for extended periods or inadvertent exposure to fish, and the severe or fatal reactions resulting from accidental exposure pose a grave risk for individuals with fish allergy [3]. The components inducing allergic reactions are mostly immunoglobulin (Ig) E-binding proteins, which are referred to as allergens. Among the fish allergens, parvalbumin is the best characterized major allergen that has been detected in several species [4–6]. In a previous study, we identified fish gelatin (type I collagen) as a potential allergen [7]. More recently, other proteins such as tropomyosin, enolases, and aldolases have also been identified as potential fish allergens [8]. Animal models of fish allergy that specifically focus on the management of fish allergenicity will serve as useful tools for improving the quality of life for individuals with fish allergy.

Animal models are beneficial for food allergy testing as they allow rapid and detailed evaluation of allergenicity of certain food products [9]. Canine species are under extensive investigation as useful models in the study of IgE-mediated hypersensitivity as they produce specific IgE to crude extracts and encounter positive oral challenges similar to those observed in human subjects [10–12]. This could be attributed to the fact that similar to that in humans, allergies in canines develop naturally following environmental exposure to a broad spectrum of allergens, including those present in food products [13, 14]. Allergic diseases in canines include atopic dermatitis, gastroenteric inflammation, and anaphylaxis [15, 16]. Owing to the numerous similarities between canine and human atopic dermatitis [17], atopic dogs have served as animal models for food allergies to cow’s milk [11], corn [18], and nuts [12].

The allergens present in food consumed by atopic dogs are assumed to correspond to those in humans [19]. However, Kubota et al. reported that IgE reactivity to allergens in dogs was different from that in humans [20]. To utilize atopic dogs as suitable models for food allergy in humans, it is necessary to analyze the homologies in the IgE reactivity in food allergy between humans and dogs. Cod is one of the most commonly consumed fish species in Europe and Japan [3], and is also the most well-characterized fish species with allergen components [8]. Nevertheless, there is limited information available on IgE reactivity to cod in dogs. Here, we investigated IgE reactivity to crude extracts and purified allergens derived from Pacific cod (*Gadus macrocephalus*) in atopic dogs.

## Results

### Clinical characteristics of atopic dogs with cod allergy

The study was conducted on 179 atopic dogs of 34 breeds, and there were 79 males and 100 females (age, 3.9 ± 3.2 years; range, 2 months–11 years). The elimination diet trials improved the atopic symptoms in 144 dogs (80%). Among the 31 atopic dogs that underwent cod oral provocation analysis, the clinical symptoms worsened after cod meat exposure in 27 atopic dogs (i.e., dogs with cod allergy). The mean age of dogs with cod allergy was 3.4 ± 2.6 years (range, 2 months–8 years), which included 12 males and 15 females. Among these, 8 dogs were administered grilled cod meat and 19 dogs were administered cod-containing dog food. The clinical responses to the provocation test included skin symptoms observed in all dogs (pruritus: 27, erythema: 16, and urticaria: 3) and concurrent gastrointestinal signs in 3 dogs (11.1%: vomiting: 3 and diarrhea: 1). Among the 27 dogs with cod allergy, 18 dogs exhibited symptoms within 3 h, whereas 9 dogs exhibited symptoms after a few days. Among the nine dogs with late-onset reaction, one dog exhibited vomiting and diarrhea.

### IgE reactivity to crude cod extracts among atopic dogs

**Fig. 1:**
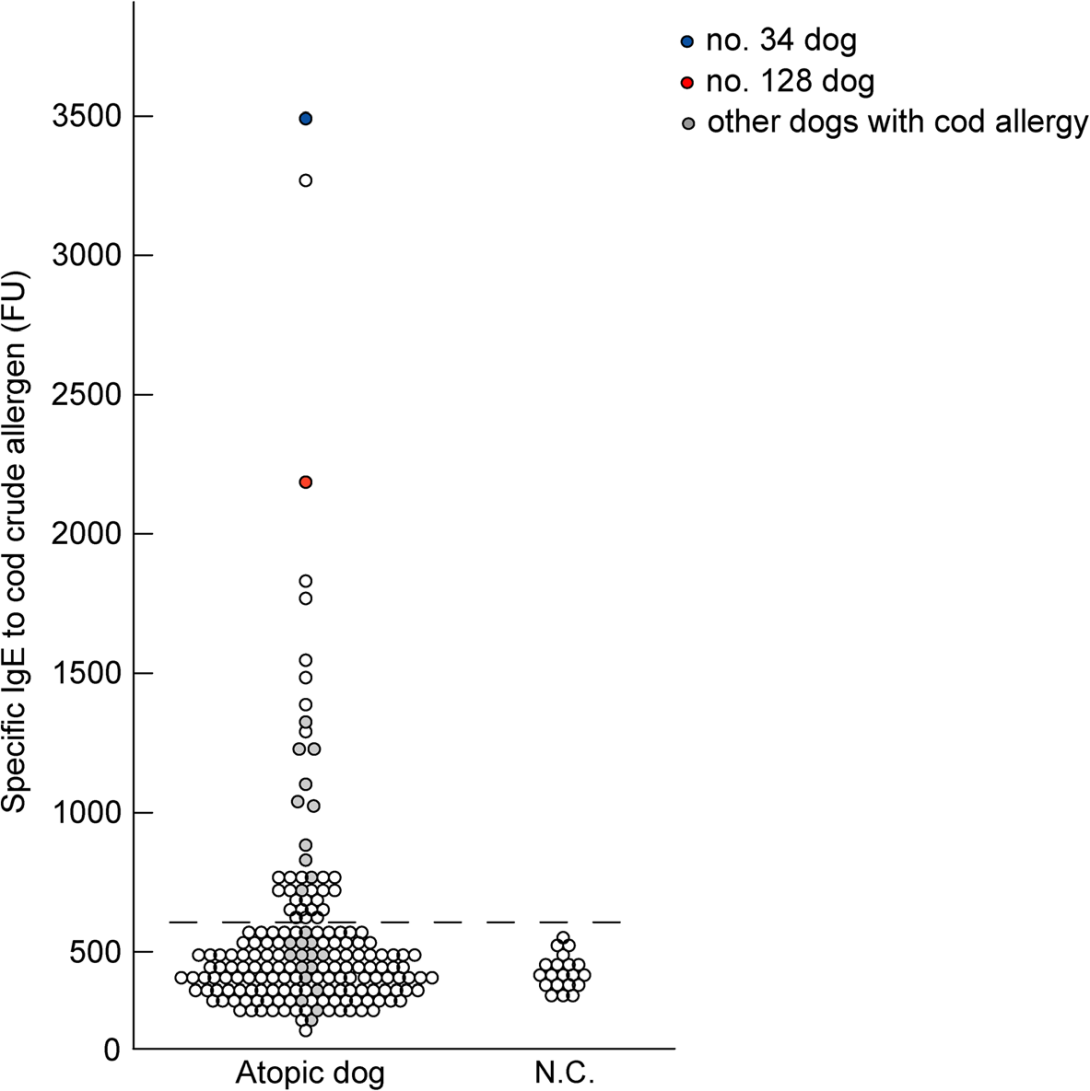
IgE reactivity to cod crude extracts in atopic dogs. Based on the levels of specific IgE to crude cod extracts in 20 negative controls, the cutoff value (mean + 3 standard deviation (SD)) was 638 fluorescence units (FU), as indicated by the dotted line. The mean FU ± SD value of IgE reactivity to crude cod extracts in 179 atopic dogs was 457 ± 61 FU. Thirty-six atopic dogs exhibited specific IgE reactivity to crude cod meat, with IgE levels ranging from 640 to 3483 FU. The blue circle indicates IgE reactivity in dog no. 34. The red circle indicates IgE reactivity in dog no. 128. The gray circles indicate IgE reactivity in other dogs with cod allergy. IgE, immunoglobulin E

Specific IgE reactivity to crude cod extracts was examined in the atopic dogs using ELISA. Twenty percent (36/179) of the dogs had increased levels of specific IgE to crude cod extract (Fig. 1). Among the atopic dogs that exhibited positive reactions in the food elimination trials, the levels of specific IgE to crude cod extracts increased in 25% (36/144). Among the dogs with cod allergy, 44% (12/27) exhibited positive IgE reactivity to crude cod extracts (Fig. 1). All the dogs with late-onset reaction exhibited negative IgE reactivity to crude cod extracts (9/9). Among the four dogs without cod allergy, one dog exhibited false-positive specific IgE reactivity to crude cod extracts.

### IgE reactivity to cod parvalbumin and collagen in atopic dogs exhibiting IgE reactivity to crude cod extracts

**Fig. 2:**
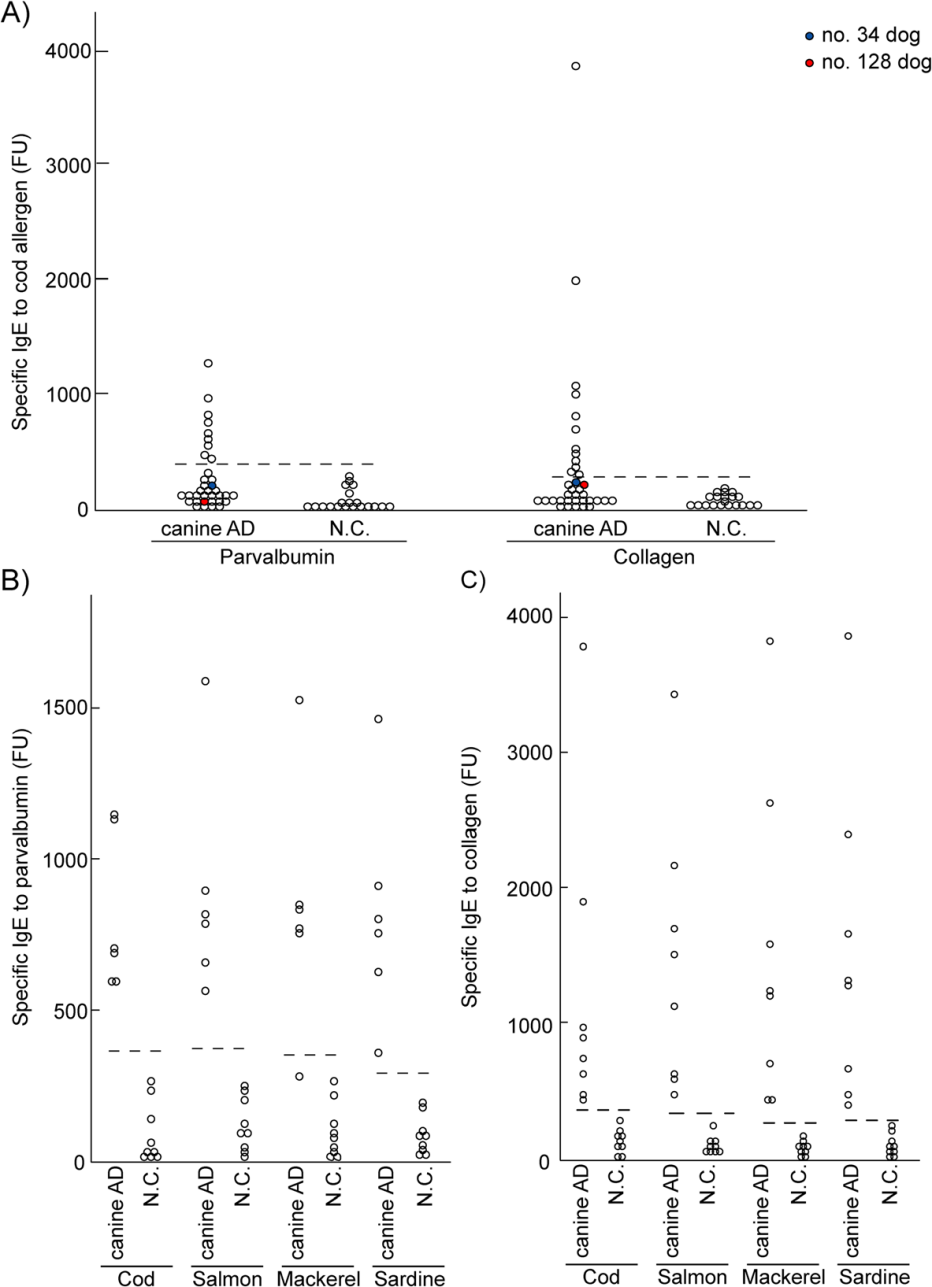
IgE reactivity to parvalbumin and collagen in atopic dogs with significantly elevated cod-specific IgE levels. **a** IgE reactivity to cod parvalbumin and collagen in 36 dogs with IgE reactivity to crude cod extract was determined using diluted sera (1:10). The dotted line indicates the cutoff value, which was calculated using sera from 20 N.C. Based on the levels of specific IgE to parvalbumin and collagen in the N.C. (mean ± SD, 84 ± 67 and 74 ± 25 FU, respectively), the cutoff values (mean + 3SD) for specific IgE against parvalbumin and collagen were 286 and 149 FU, respectively. The levels of specific IgE to parvalbumin and collagen in atopic dogs were 299–1121 and 151–3774 FU, respectively. The blue circle represents IgE reactivity in dog no. 34. The red circle represents IgE reactivity in dog no. 128. The levels of **b** Parvalbumin-specific IgE and **c** collagen-specific IgE was measured in the sera of six dogs with specific IgE to parvalbumin and eight dogs with specific IgE to collagen and in those of nine healthy dogs (N.C.). The cutoff values (mean + 3SD) of specific IgE were 379, 374, 351, and 372 FU for parvalbumin from cod, salmon, mackerel, and sardine, respectively, which are indicated by the dotted lines. The cutoff values (mean + 3SD) of specific IgE were 393, 310, 231, and 372 for collagen from cod, salmon, mackerel, and sardine, respectively. N.C., negative control

IgE reactivity to the purified cod allergens parvalbumin and collagen was tested in the sera of the 36 dogs with IgE reactivity to crude cod extracts using ELISA. IgE reactivity to parvalbumin was observed in 25% (9/36), whereas IgE reactivity to collagen was observed in 33% (12/36) of the dogs. IgE reactivity to both parvalbumin and collagen was observed in 16% (6/36) (Fig. 5) of the dogs. However, 58% (21/36) of the dogs did not display IgE reactivity to the purified cod allergens (Fig. 2a), which indicates that the reactivity was not specific to fish proteins. We also compared the IgE reactivity to parvalbumin and collagen derived from four fish species (cod, salmon, mackerel, and sardine) in six dogs that reacted to cod parvalbumin and eight dogs that reacted to cod collagen. In these dogs, IgE reactivity to parvalbumin was observed upon reaction with samples derived from cod, salmon, and sardine, while parvalbumin derived from mackerel did not induce a reaction (Fig. 2b). Meanwhile, IgE reactivity to collagen derived from all the fish species was observed in 100% (8/8) of the dogs (Fig. 2c).

### Identification of other cod allergen components in cod allergy in atopic dogs

**Fig. 3:**
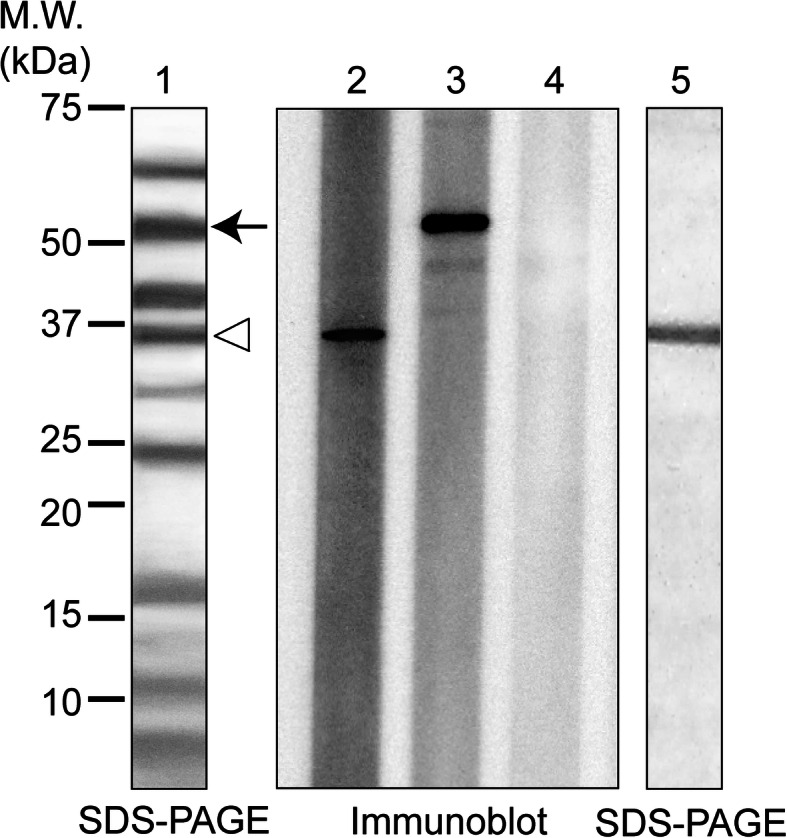
Immunoblotting for crude cod extracts. In the left column, the molecular standard is shown. Lanes 1 and 5 were stained by Coomassie brilliant blue R250. Lanes 2 and 3 depict immunoblotting in the sera samples of two atopic dogs (lane 2, dog no. 34; lane 3, dog no. 128); lane 4 represents the serum of a healthy dog used as negative control. The arrow and arrowhead next to lane 1 indicate the bands analyzed by liquid chromatography-tandem mass spectrometry, which corresponded to the band detected by immunoblotting (immunoblotting in lanes 2 and 3)

Among the 20 atopic dogs with IgE sensitivity to crude cod extracts that did not display reactivity to parvalbumin or collagen, the sera from two dogs (no. 34 and no. 128) were collected, which provided sufficient quantity of sera. While the samples showed reactivity to crude cod extract, no reactivity was observed upon treatment with cod parvalbumin or collagen, as confirmed by the provocation test using cod-containing foods (See Supplementary Table 1, Additional File 1). After the separation of the crude cod extract proteins using SDS-PAGE, IgE-reactive protein bands formed at approximately 35 and 55 kDa were observed in the IgE immunoblotting experiments (Fig. 3). LC-MS/MS, which was performed to identify the corresponding proteins in SDS-PAGE, revealed that the protein band of approximately 35 kDa corresponded to tropomyosin derived from golden gray mullet (*Liza aurata*), and the protein band of approximately 55 kDa corresponded to α enolase derive from python (*Python regius*) (Table 1).

**Table 1:** Suspected allergens detected in two dogs with IgE reactivity to crude cod extracts using LC-MS/MS

Band (kDa)	Accession number	Protein name/species	Theoretical		LC-MS/MS analysis		
			MW (kDa)	PI	Score	Peptide matches	% coverage
35	P84335	Tropomyosin/*Liza aurata*	32.710	4.69	111	3	7
55	gi17367183	Alpha enolase/*Python regius*	47.541	6.97	64	1	2

*LC-MS/MS* liquid chromatography-tandem mass spectrometry, *MW* molecular weight, *PI* isoelectric point

We next purified cod tropomyosin from the crude cod extract and confirmed its identity using SDS-PAGE (Fig. 3). The levels specific IgE to fish tropomyosin in the serum of dog no. 34 were measured using ELISA (See Supplementary Figure 2, Additional File 1); the results revealed that the levels were significantly higher than those in the sera of the 20 control dogs. The levels of specific IgE to tropomyosin derived from other fish species were also higher in dog no. 34 compared to those in the negative controls.

Dog no. 34 presented with specific IgE and a positive reaction in the intradermal test for crude mite extract (House dust mite; *Dermatophagoides farinae*) (data not shown). We evaluated the levels of specific IgE to recombinant mite tropomyosin (*Der f* 10) in the serum of dog no. 34 using ELISA (See Supplementary Figure 1, Additional File 1) and observed that the levels were significantly higher than those in the sera of the 20 control dogs.

### IgE reactivity to cod tropomyosin and crude cod extract in atopic dogs

**Fig. 4:**
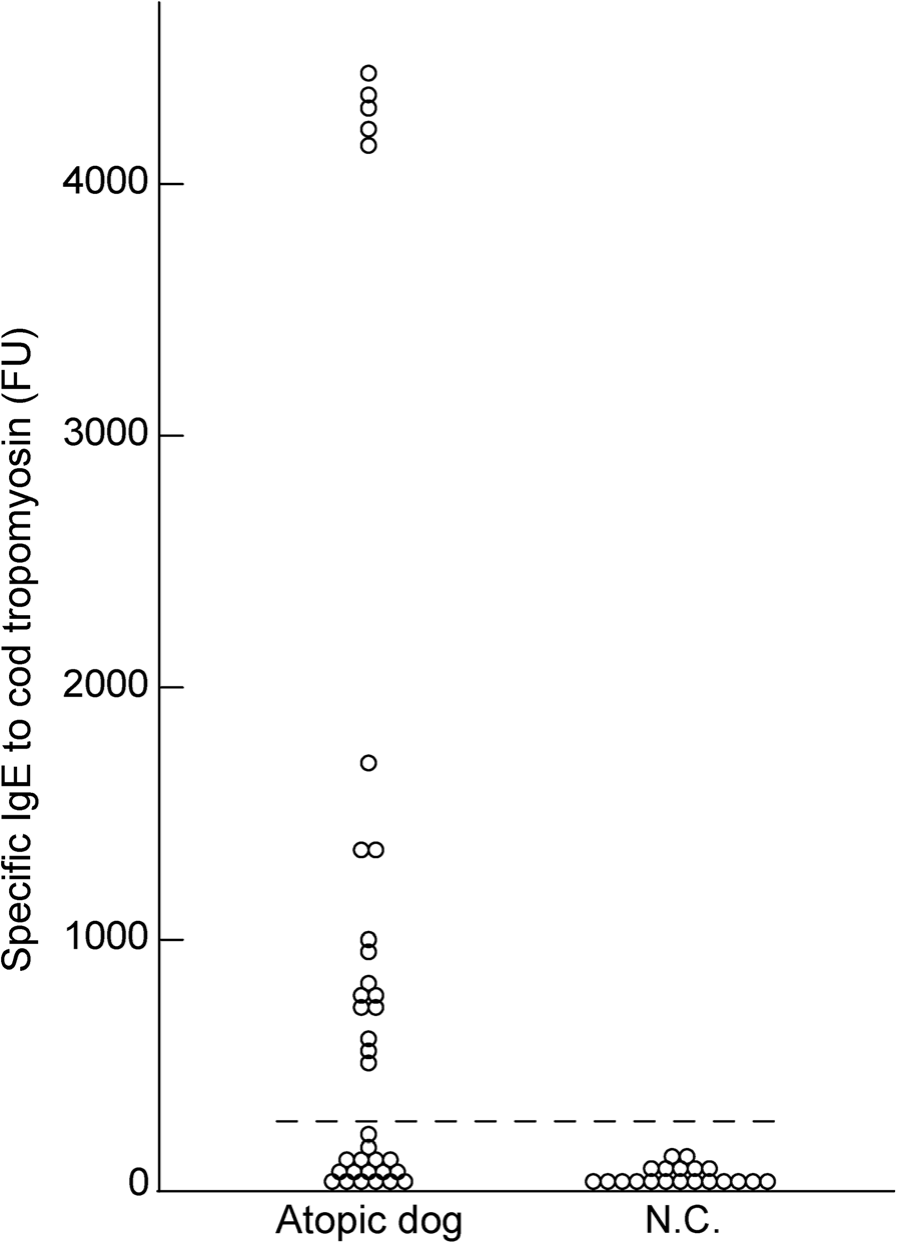
Reactivity to cod tropomyosin in dogs with specific IgE to crude cod extracts using ELISA. The cutoff value (dotted line) calculated from 20 negative control samples. Based on the levels of specific IgE to tropomyosin in the negative controls (mean ± SD, 33 ± 67 FU), the cutoff value (mean + 3SD) of IgE reactivity in 36 atopic dogs with specific IgE to crude cod extracts was determined as 234 FU. ELISA, enzyme-linked immunosorbent assay

**Fig. 5:**
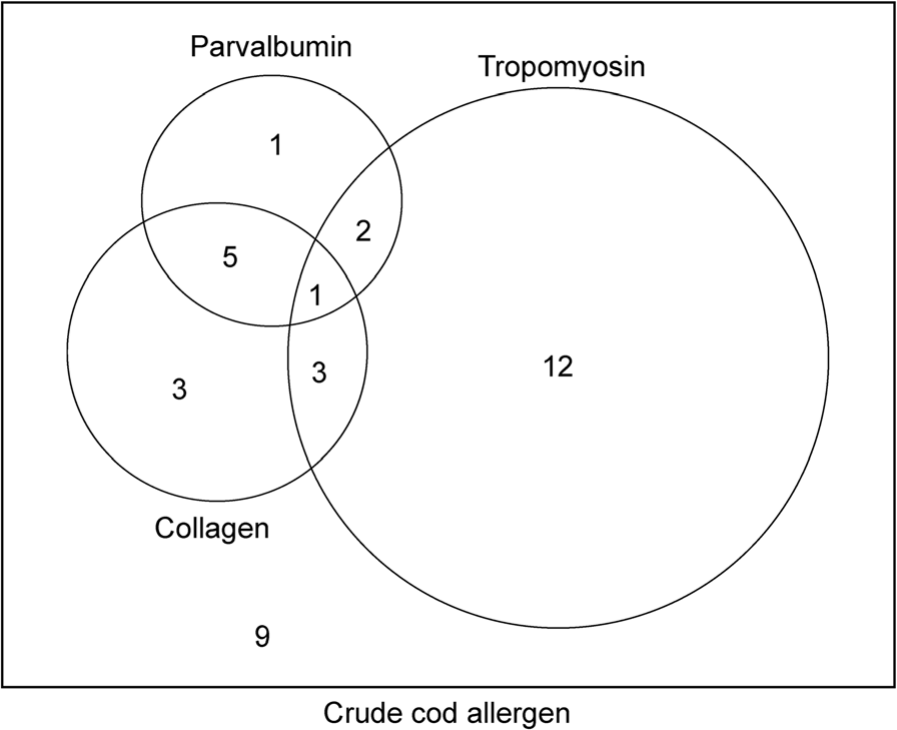
Venn diagram of the number of dogs harboring fish allergen-specific IgE

Using the sera of 36 atopic dogs with IgE reactivity to cod extract, we determined the IgE reactivity to cod tropomyosin with ELISA (Fig. 4), and observed that 50% (18/36) of the dogs exhibited IgE reactivity to tropomyosin. As shown in Fig. 5, 67% (12/18) of the dogs with atopic dermatitis that had high levels of specific IgE to crude cod extract and tropomyosin did not have specific IgE to cod parvalbumin or collagen. Meanwhile, 25% (9/36) of the dogs did not display IgE reactivity to any of the tested allergens.

## Discussion

Atopic dermatitis affects approximately 10–20% of the canine population [21]; hence, the canine atopic dermatitis model could be useful as a spontaneous animal model that can be acquired easily. To assess the potential of atopic dogs with fish allergy as animal models, characterization of the IgE reactivity in dogs with cod allergy is useful. We reported the prevalence rate, allergenicity to individual components, and symptomatic association in dogs with cod allergy.

The prevalence of fish allergy might be higher than expected. The rate of food allergy or intolerance to fish has previously been reported to be 1.3% (4/297) in dogs with food allergy or intolerance that was diagnosed by food trials and provocation tests [22]. However, the present study revealed that 20% (36/179) of the dogs exhibited increased levels of specific IgE to crude cod extract and 25% (36/144) of dogs had food allergy or intolerance, as indicated by the findings of the food elimination trials (Fig. 1). Moreover, dogs in Japan might be exposed to fish at a higher frequency than dogs in other countries such as USA. Our field survey on commercial canine dry food products for the estimation of the difference in fish allergen exposure between dogs from Japan and other countries revealed that 75% (117/157) of the Japanese canine dry food products contained fish. In contrast, only 9% (7/82) of the products produced in Australia and the USA contained fish. These evidences suggest that atopic dogs in Japan might be at a higher risk of developing fish allergy owing to the higher frequency of dietary exposure to fish. These characteristics of atopic dogs might mimic those of humans.

To the best of our knowledge, this is the first study to determine the allergenic potency of parvalbumin and collagen in dogs in terms of their capacity to induce the subsequent production of allergen-specific IgE antibodies. Parvalbumin has a higher allergenic potency than collagen in humans with cod allergy [2]. The present study revealed that the rate of collagen allergy was higher than that of parvalbumin in dogs (Fig. 5) and collagen induced a stronger reactivity than that induced by parvalbumin based on the levels of specific IgE in these animals (Fig. 2a). This discrepancy might be attributed to the degradation of parvalbumin in dog food during the physical and chemical steps in food processing, since parvalbumin is a water-soluble protein, unlike collagen [4, 23]. Additionally, humans exhibit broad cross-reactivity to parvalbumin and collagen from distinct fish species [3, 24] and the same might occur in dogs with fish allergy as well. The current study suggested that the IgE reactivity to collagen and parvalbumin derived from cod in dogs was comparable to the reactivity to those derived from other fish species such as salmon, mackerel, and sardine (Fig. 2b and c).

Of note, the rate of tropomyosin allergy was higher than those of parvalbumin or collagen allergies in dogs (Fig. 5) and the levels of specific IgE to tropomyosin was higher than those to other allergenic components derived from cod, such as parvalbumin and collagen, in dogs with cod allergy (Figs. 2a and 4). Tropomyosin was shown to be a fish allergen in a study using the sera of human patients with tilapia allergy [25]. Additionally, our comparison of the protein sequence of tropomyosin derived from cod to those derived from other commercial fish species revealed a sequence similarity of 94–99% between the different types of tropomyosin (See Supplementary Table 2, Additional File 1). Tropomyosin is a major allergen that has been attributed in crustacean allergy [26] as well as mite allergy [27] in humans. Although a comparison of the tropomyosin protein sequence from cod and shrimp revealed a low sequence similarity between these (See Supplementary Table 2, Additional File 1), fish-shrimp cross-reactivity has been reported previously in humans [28, 29]. Additionally, mite-crustacean cross-reactivity has been reported widely in humans, and the sequence similarity of tropomyosin derived from the two species is over 90% [30]. In dogs, mites are one of the most frequently sensitizing allergens, and mite tropomyosin is the allergenic component from mites in canine atopic dermatitis [31]. Additionally, in this study, the serum of dog no. 34 elicited reactivity to mite and cod tropomyosin at comparable levels (See Supplementary Figure 1, Additional File 1). Collectively, these findings suggest that mite allergy in dogs with IgE reactivity to mite tropomyosin might be associated with increased IgE reactivity to cod tropomyosin.

The findings of the current study also suggest that enolase could serve as a potential allergen associated with canine fish allergy (Fig. 3 and Table 1). Enolase was recently defined as a fish allergen that exhibited cross-reactivity with chicken in humans and dogs [29, 32]. Numerous fish proteins, apart from the purified proteins that are recognized as critical allergenic components in humans, have been registered in the International Union of Immunological Societies allergen database [33]. In some cases, minor allergens in humans can act as major allergens in dogs [20, 34]. Moreover, in the present study, 25% (9/36) of the dogs with specific IgE to crude cod extracts did not exhibit IgE reactivity to any of the three major purified allergens in ELISA, which implies that other cod allergens be responsible for inducing fish allergy in dogs. Future studies should focus on the identification of other allergens using the sera of atopic docs with IgE reactivity to cod extracts.

Atopic dermatitis with food allergy can be a manifestation of an IgE- or a non-IgE-mediated reaction to food products in humans [35, 36]. Similarly, IgE- and non-IgE mediated reactions might also occur in atopic dogs. Approximately 10% of atopic dogs exhibited positive results in the lymphocyte stimulation test to fish [37, 38]. Lymphocyte stimulation tests are used for the diagnosis of non-IgE-mediated food allergies in humans [39, 40]. Moreover, non-IgE mediated cod allergies may be more common in dogs than in humans. Among dogs with cod allergy, the rate of IgE reactivity to crude cod extracts (44%; Fig. 1) was lower than that in humans (90–95%) [41]. In 60 % of dogs with cod allergy that did not exhibit IgE reactivity to crude cod extracts, clinical reactions were observed after a few days in the oral provocation tests. Conversely, fish allergy in humans generally presents with the classic symptoms of food allergy shortly after fish intake [3, 41]. These characteristics of non-IgE mediated reactions in atopic dogs might be independent of those in humans.

## Conclusions

The present study suggests that although IgE reactivities in cod allergy in dogs and humans share some aspects, certain differences remain. Therefore, atopic dogs can serve as animal models of cod allergy if the experiments are conducted with adequate caution. Further analysis would be required to characterize cod allergy in dogs. As this experiment was an illustration, it was not feasible to examine the relationship between the detailed clinical symptoms and responsiveness to each allergen using methods such as specific IgE component analysis. We aim to focus on analyzing the aforementioned relationship in future studies.

## Methods

### Sera of atopic dogs and controls

To examine IgE reactivity in atopic dogs, we collected surplus sera from 179 dogs that were clinically diagnosed with atopic dermatitis from among dogs visiting the Fujimura Animal Hospital (Osaka, Japan) based on the criteria proposed by Willemse [42] and Prelaud et al. [43], which is clinically compatible with the symptoms of atopic dermatitis in humans [17]. Twenty samples collected from laboratory dogs were used as negative controls. The dogs were housed indoors as experimental laboratory animals and did not have prior exposure to fish antigens. None of the laboratory dogs exhibited signs of atopic dermatitis. All sera samples were stored at − 80 °C until used. Verbal informed consent was provided by the dog owners. All experimental procedures were performed in accordance with Japanese law and were approved by the animal care and user committee of Azabu University.

### Food elimination and oral provocation tests

The elimination diet trials were performed for 6–8 weeks using commercial hydrolyzed diets on 179 dogs that exhibited clinical symptoms of atopic dermatitis. The dog owners fed commercial hydrolyzed diets that contained ingredients that the dog had no prior exposure to. The ingredients were confirmed based on information provided by the dog owners. When clinical signs were resolved (disappearance of pruritus and hair regrowth), food provocation was performed for 1 week by administering the original diet. The dogs were checked for clinical symptoms by a veterinary physician every 2 weeks or once per month. Among the 144 dogs that exhibited clinical improvement in response to the food elimination trials, 31 dogs were observed to exhibit cod reactivity, as evidenced by the oral provocation tests. These dogs were selected based on the diet history, admitted to the animal hospital, and were challenged with various food products containing cod components, including grilled cod meat and cod-containing dog foods (Select Protein Cod and Rice Dry, Royal Canin, Aimargues, France). The provocation trials were conducted by one of the authors (MF), following which the complete resolution of the clinical signs occurred after conducting the food elimination tests. The cod provocation tests were discontinued immediately upon the relapse of the clinical signs that included vomiting, diarrhea, erythema, pruritic, urticaria, and conjunctival hyperemia. We obtained verbal consent for the food elimination trials and provocation tests from the dog owners, as previously described [37]. After the procedures, all dogs were returned to their owners. Supplementary Figure 1, Additional file 1 presents the flow diagram for study participants.

### Preparation of crude cod extracts

Pacific cod was purchased from a fish market in Japan. The fresh, raw meat of four fishes (500 μg) was homogenized in 500 μl of phosphate-buffered saline (PBS, 10 mM pH 7.2) overnight at 4 °C under rotating conditions. After centrifugation at 21500 x *g* for 5 min at 4 °C, the supernatant was collected, and the protein was quantified using the BCA protein assay (Bio-Rad, Hercules, CA, USA).

### Purification of parvalbumin, collagen, and tropomyosin

Fish parvalbumin [4] and collagen [23] were purified as described previously. Tropomyosin was purified from the freeze-dried powder using Bailey’s method with slight modifications [44]. Briefly, freeze-dried fish powder was stirred in a beaker with 75 ml extraction buffer containing 15 mM Tris HCl pH 7.6 (Sigma Aldrich, St Louis, MO, USA), 1 M KCl (Kanto Kagaku, Tokyo, Japan), and 2 mM dithiothreitol (Sigma Aldrich) overnight at 4 °C. The extract was collected by centrifugation at 5400 x *g* for 10 min at 4 °C. The pH of the supernatant was adjusted to 4.5 using 1 N HCl to precipitate tropomyosin, and the precipitate was collected by centrifugation at 5400 x *g* for 10 min at 4 °C. The isoelectric precipitation process was repeated once and the precipitate was dissolved in the extraction buffer. The supernatant obtained after the extraction was collected by centrifugation and fractionated using ammonium sulfate to a concentration of 50%. The sample precipitated by ammonium sulfate fractionation was dissolved and dialyzed against PBS. The obtained protein extracts were identified by sodium dodecyl sulfate (SDS)-polyacrylamide gel electrophoresis (PAGE).

### SDS-PAGE and immunoblotting

SDS-PAGE was performed according to the method described by Laemmli [45]. Precision plus protein standards (Bio-Rad, Hercules, CA, USA) were used as molecular-mass markers. The crude cod extracts were separated electrophoretically using 5–20% gradient polyacrylamide gels, and the proteins were visualized either by Coomassie brilliant blue R250 (Bio-Rad) staining or by transferring onto polyvinylidene difluoride membranes (GE Healthcare, Chicago, IL, USA). Immunoblotting was performed as described previously [7]. IgE from patient dog sera were used as primary antibodies, and were diluted at a 1:10 ratio in Tris buffered saline containing 0.1% Tween-20 and 5% nonfat dried milk (pH 7.4). Mouse monoclonal anti-dog IgE antibodies (0.5 μg/ml, clone D9) were used as secondary antibodies [46]. Horseradish peroxidase-conjugated goat anti-mouse IgG (0.05 μg/ml) (Bio-Rad Laboratories) were used as tertiary antibodies. An enhanced chemiluminescence immunoblotting detection reagent (GE Healthcare) and the ImageQuant LAS 4000mini (GE Healthcare) were used for detection and visualization, respectively.

### Liquid chromatography-tandem mass spectrometry (LC-MS/MS)

For LC- MS/MS, the protein bands formed after the immunoblotting experiment were excised, and in-gel digestion was performed using 0.5 mg N-tosyl-L-phenylalanine chloromethyl ketone-treated trypsin (Promega, Madison, Wisconsin) at 37 °C for 16 h. The tryptic digests were acidified using formic acid (pH < 2.0) and centrifuged at 21500 x *g* for 15 min. The supernatants were analyzed using a high-performance liquid chromatograph (Advance System; AMR, Tokyo, Japan) connected to an electrospray ionization triple quadrupole mass spectrometer (4000 QTRAP; AB Sciex, Framingham, MA, USA). The extracts were injected into a reversed-phase column (electrospray ionization column [octa decyl silyl]; particle inner diameter, 75 mm; length, 100 mm; diameter 3 mm; LC Assist, Tokyo, Japan) that was eluted with a 5–45% acetonitrile gradient containing 0.1% formic acid for 60 min at 300 nl/min. Ionization was performed at an ion-spray voltage of 2000 V and a capillary temperature of 200 °C. The mass spectrometer was operated in the positive ion mode between 450 and 1200 m/z. The MS/MS spectra were obtained in the enhanced production scan mode, and two higher-intensity peaks in each mass spectrometry scan were selected for collision-induced dissociation.

The MS/MS data were used to search the entries under the *Liza aurata* category in the UniProt database with the Mascot peptide search engine. An MS tolerance of 1.0 Da for precursor ion and an MS/MS tolerance of 0.8 Da were set as the windows of processing parameters for matching the peptide mass values.

### Fluorometric enzyme-linked immunosorbent assay (ELISA) for the detection of allergen-specific serum IgE

The levels of specific IgE to cod crude extracts and individual purified allergens were measured using fluorometric ELISA, as previously described [20]. A microplate (NUNC Immuno Plate Maxisorp F96; Nalge Nunc International, Roskilde, Denmark) was coated with the crude cod extracts (10 μg/ml) or the purified allergens (parvalbumin, collagen, or tropomyosin; 1 μg/ml) and maintained overnight at 4 °C. After washing, the plate was incubated with diluted sera (1:10) in PBS with 10% (v/v) fetal calf serum and 0.05% (v/v) Tween 20 at room temperature for 3 h. The plate was then washed and incubated with mouse monoclonal anti-dog IgE antibody (0.5 μg/ml, clone D9) overnight at 4 °C [46]. After washing with PBS containing 0.1% (v/v) Tween 20 (PBS-T), the plate was incubated with biotinylated rat monoclonal anti-mouse IgG1 (Zymed Laboratories, San Francisco, CA, USA) for 1 h at room temperature. After washing, the plate was incubated with β-D-galactosidase-conjugated streptavidin (Zymed Laboratories) for 1 h at room temperature. After the final wash, the plate was incubated with 0.1 mM 4-methylumbelliferyl-β-D-galactopyranoside (Sigma Aldrich) for 2 h at 37 °C. The enzymatic reaction was terminated by adding 0.1 M glycine-NaOH (pH 10.2). The fluorescence intensity was measured in terms of fluorescence units (FU) using a microplate fluorescence reader (Fluoroskan; Flow Laboratories, McLean, VA, USA). The absorbance was measured at 355 nm using a 460 nm reference filter. All the washing steps were performed thrice for 5 min in PBS-T. The cutoff value was determined as the average + three standard deviations (SDs) of FU in the sera from the 20 dogs that served as negative controls in the analysis of the levels of specific IgE to cod crude extracts. ELISA for parvalbumin or collagen from salmon (Atlantic salmon; *Salmo salar*), sardine (Japanese pilchard; *Sardinops melanostictus*), and mackerel (Chub mackerel; *Scomber japonicus*) were performed using individual sera, which ensured that a substantial quantity of sera was available (See Supplementary Figure 1, Additional File 1). All tests were performed in triplicate**.**

## Supplementary information

**Additional file 1: Supplementary Data. Table S1.** Clinical characteristics of dogs with IgE reactivity to crude cod extracts. **Table S2.** Amino acid sequence homology of tropomyosin from different species and from *Liza aurata* using BLASTP. **Figure S1.** Flow diagram for study participants. **Figure S2.** IgE reactivity to tropomyosin from four different fishes and house dust mite (dog no. 34). **Figure S3.** Non-cropped, non-modified images (A) of the Coomassie blue-stained SDS-PAGE and (B) of immunoblotting for crude cod extracts.

## Data Availability

All data generated or analyzed during this study are included in this published article and its supplementary information files.

## Abbreviations

AD: Atopic dermatitis
ELISA: Enzyme-linked immunosorbent assay
FU: Fluorescence units
Ig: Immunoglobulin
LC: Liquid chromatography
MS/MS: Tandem mass spectrometry
PBS: Phosphate buffered saline
SD: Standard deviation
SDS-PAGE: Sodium dodecyl sulfate Poly-acrylamide gel electrophoresis
