# Supplementary material for: IgE reactivity to fish allergens from Pacific cod (Gadus macrocephalus) in atopic dogs
Source: BMC Vet Res. 2020 Sep 16;16:341. doi: 10.1186/s12917-020-02559-1 (PMC7493343; doi:10.1186/s12917-020-02559-1)
Supplement: Supplementary file 1 — Additional file 1: Supplementary Data. Table S1. Clinical characteristics of dogs with IgE reactivity to crude cod extracts. Table S2. Amino acid sequence homology of tropomyosin from different species and from Liza aurata using BLASTP. Figure S1. Flow diagram for study participants. Figure S2. IgE reactivity to tropomyosin from four different fishes and house dust mite (dog no. 34). Figure S3. Non-cropped, non-modified images (A) of the Coomassie blue-stained SDS-PAGE and (B) of immunoblotting for crude cod extracts. [file 12917_2020_2559_MOESM1_ESM.pdf]

Online supporting information

IgE reactivity to fish allergens from Pacific cod (*Gadus macrocephalus*) in atopic dogs

Ichiro Imanishi[1,2], Jumpei Uchiyama[1], Keijiro Mizukami[1], Junichi Kamiie[3], Keigo Kurata[4], Keita Iyori[5], Masato Fujimura[6], Kuniyoshi Shimakura[7], Koji Nishifuji[8], Masahiro Sakaguchi[1]*

[1]Laboratory of Veterinary Microbiology I, School of Veterinary Medicine, Azabu University, Kanagawa, Japan

[2]Department of Microbiology, Kitasato University School of Medicine, Kanagawa, Japan.

[3]Laboratory of Veterinary Pathology, School of Veterinary Medicine, Azabu University, Kanagawa, Japan

[4]Institute of Tokyo Environmental Allergy, ITEA Inc., Tokyo, Japan

[5]Vet Derm Tokyo, Laboratory and Clinical Dermatology Services, Tokyo, Japan

[6]Fujimura Animal Hospital, Osaka, Japan

[7]Department of Food Science and Technology, Tokyo University of Marine Science and Technology, Tokyo, Japan

[8]Laboratory of Veterinary Internal Medicine, Faculty of Agriculture, Tokyo University of Agriculture and Technology, Tokyo, Japan

Supplementary Table 1. Clinical characteristics of dogs with IgE reactivity to crude cod extracts

	Dog No.	
	34	128
Breed	Shiba Inu	French Bulldog
Sex	Female	Castrated Male
Age (year)	6	7
Onset age (year)	3	4
Allergy symptoms	Non-seasonal pruritus, erythema, lichenization, pigmentation, excoriation, and alopecia	Non-seasonal pruritus, eczema, erythema, and alopecia
Specific IgE to crude cod extract (FU)	3483	2183
Cod type used in the provocation test	Grilled cod meat	Dry food containing cod
Symptoms induced during the provocation test	Pruritus, erythema, eczema, excoriation, abdominal pain, and diarrhea	Pruritus, erythema, eczema, and excoriation

FU, fluorescence units

Supplementary Table 2. Amino acid sequence homology of tropomyosin from different species and from *Liza aurata* using BLASTP

Species	Common name	Scientific name	Accession number	AAs n	Identities %	Positives %	Gaps %
Fish	Golden gray mullet	*Liza aurata*	P84335	284			
Fish	Alaska pollack (cod)	*Gadus chalcogrammus*	BAC44994	284	97	97	0
Fish	Mozambique tilapia	*Oreochromis mossambicus*	AFV53352	284	96	97	0
Fish	Bastard halibut	*Paralichthys olivaceus*	AHN52084	284	96	98	0
Fish	Atlantic Salmon	*Salmo salar*	NP_001117128	284	95	98	0
Fish	Atlantic Bluefin Tuna	*Thunnus thynnus*	BAD01050	284	96	97	0
Fish	Japanese pufferfish	*Takifugu rubripes*	BAC10576	284	95	97	0
Shrimp	Greasy back prawn	*Metapenaeus ensis*	Q25456	274	55	76	0
Shrimp	Black tiger shrimp	*Penaeus monodon*	A1KYZ2	284	56	77	0
Mite	American house dust mite	*Dermatophagoides farinae*	Q23939	283	57	76	0
Mite	European house dust mite	*Dermatophagoides pteronyssinus*	O18416	284	57	75	0

AA, amino acid; BLASTP, the protein basic local alignment search tool

Supplementary Figure 1. Flow diagram for study participants

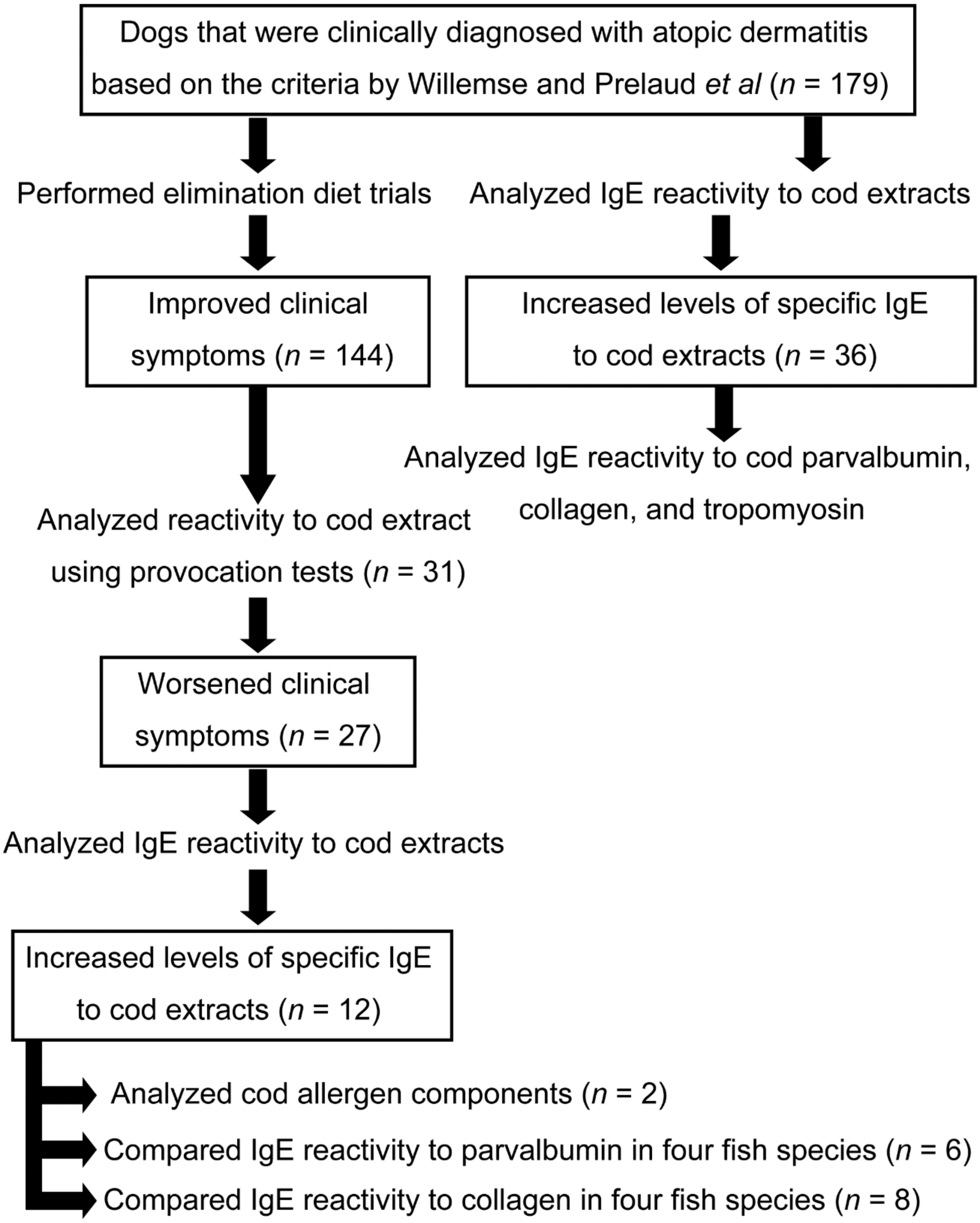

Supplementary Figure 2. IgE reactivity to tropomyosin from four different fishes and house dust mite (dog no. 34)

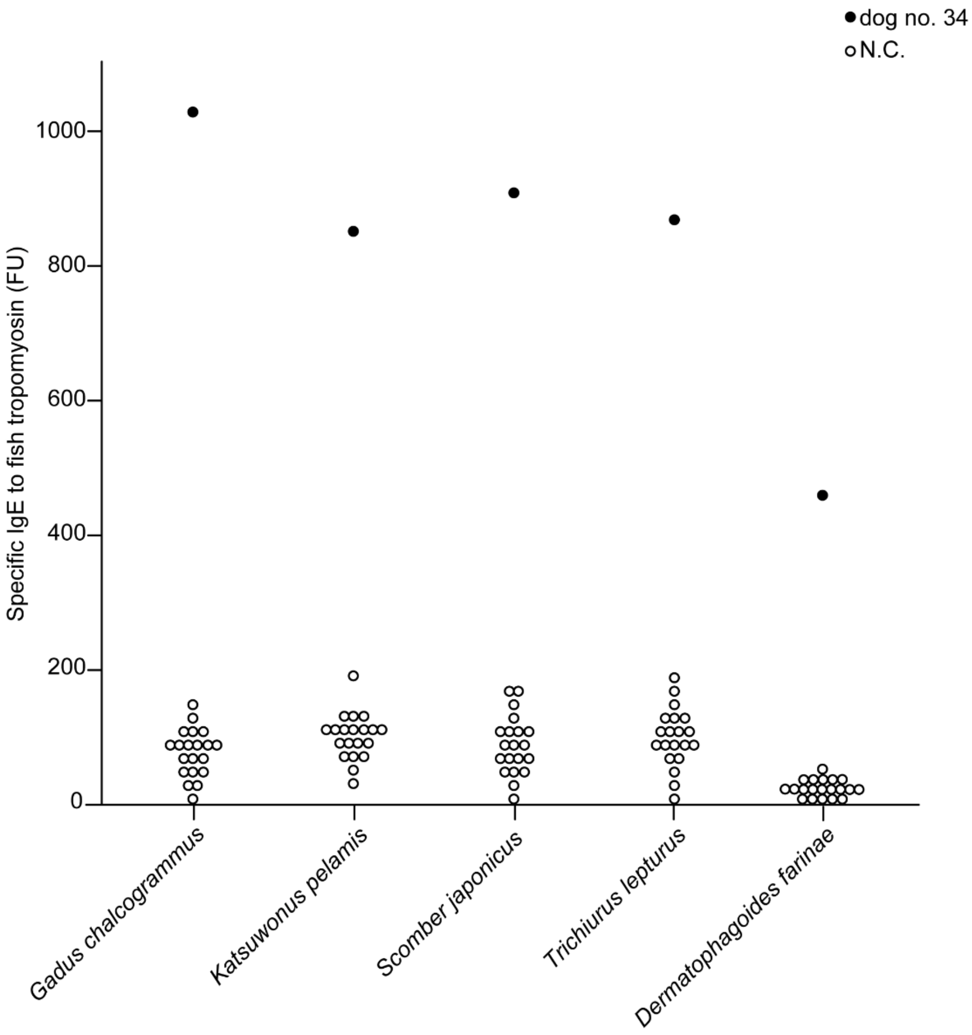

The black circles represent IgE reactivity in dog no. 34, and the white circles represent IgE reactivity in negative control samples. The mean ± 3 standard deviation (SD) levels of IgE to cod tropomyosin (*Gadus chalcogrammus*) in the sera of 20 control dogs was 67 ± 105 FU. The mean ± 3 SD levels of IgE to tropomyosin from bonito (*Katsuwonus pelamis*), sardine (*Scomber japonicus*), hairtail (*Trichiurus lepturus*), and house dust mite (*Dermatophagoides farinae*) were 88 ± 102, 91 ± 129, 79 ± 129, and 32 ± 35 FU, respectively.

Supplementary Figure 3. Non-cropped, non-modified images (A) of the Coomassie blue-stained SDS-PAGE and (B) of immunoblotting for crude cod extracts.

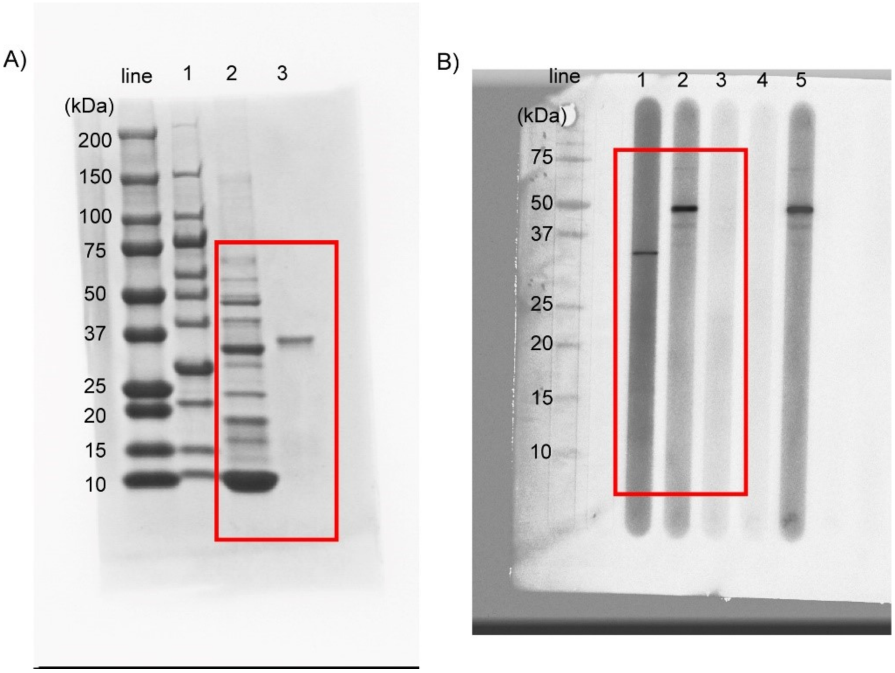

(A) Lane 1, molecular weight markers (Protein Ladder, Mixell, Hiroshima, Japan). Lane 2, ten μg of crude cod extracts stained by Coomassie brilliant blue R250. Lane 3, three μg of cod tropomyosin stained by Coomassie brilliant blue R250. (B) Lanes 1, 2, 5 depict immunoblotting in the sera samples of two atopic dogs (lane 1, dog no. 34; lane 2 and 5, dog no. 128); lanes 3 and 4 represents the serum of a healthy dog used as negative control. Representative blots were presented in Figure 3 (areas delineated in red frames).
